# Somatic Mutations in the Chromatin Remodeling Gene *ARID1A* Occur in Several Tumor Types

**DOI:** 10.1002/humu.21633

**Published:** 2011-10-18

**Authors:** Siân Jones, Meng Li, D Williams Parsons, Xiaosong Zhang, Jelle Wesseling, Petra Kristel, Marjanka K Schmidt, Sanford Markowitz, Hai Yan, Darell Bigner, Ralph H Hruban, James R Eshleman, Christine A Iacobuzio-Donahue, Michael Goggins, Anirban Maitra, Sami N Malek, Steve Powell, Bert Vogelstein, Kenneth W Kinzler, Victor E Velculescu, Nickolas Papadopoulos

**Affiliations:** 1 Ludwig Center for Cancer Genetics and Therapeutics and Howard Hughes Medical Institute, Johns Hopkins Kimmel Cancer CenterBaltimore, Maryland; 2 Texas Children's Cancer Center and Departments of Pediatrics and Molecular and Human Genetics, Baylor College of MedicineHouston, Texas; 3 Department of Pathology, Netherlands Cancer Institute/Antoni van Leeuwenhoek Hospital Plesmanlaan 121Amsterdam, The Netherlands; 4 Department of Medicine, and Seidman Cancer Center at Case Western Reserve University and Case Medical Center of University Hospitals of ClevelandCleveland, Ohio; 5 Department of Pathology, Pediatric Brain Tumor Foundation, and Preston Robert Tisch Brain Tumor Center at Duke University Medical CenterDurham, North Carolina; 6 Department of Pathology, The Sol Goldman Pancreatic Cancer Research Center, Johns Hopkins Medical InstitutionsBaltimore, Maryland; 7 Division of Hematology and Oncology, Department of Internal Medicine, University of MichiganAnn Arbor, Michigan; 8 Division of Gastroenterology, Department of Internal Medicine, University of Virginia Health SystemCharlottesville, Virginia

**Keywords:** ARID1A, cancer, chromatin remodeling

## Abstract

Mutations in the chromatin remodeling gene *ARID1A* have recently been identified in the majority of ovarian clear cell carcinomas (OCCCs). To determine the prevalence of mutations in other tumor types, we evaluated 759 malignant neoplasms including those of the pancreas, breast, colon, stomach, lung, prostate, brain, and blood (leukemias). We identified truncating mutations in 6% of the neoplasms studied; nontruncating somatic mutations were identified in an additional 0.4% of neoplasms. Mutations were most commonly found in gastrointestinal samples with 12 of 119 (10%) colorectal and 10 of 100 (10%) gastric neoplasms, respectively, harboring changes. More than half of the mutated colorectal and gastric cancers displayed microsatellite instability (MSI) and the mutations in these tumors were out-of-frame insertions or deletions at mononucleotide repeats. Mutations were also identified in 2–8% of tumors of the pancreas, breast, brain (medulloblastomas), prostate, and lung, and none of these tumors displayed MSI. These findings suggest that the aberrant chromatin remodeling consequent to *ARID1A* inactivation contributes to a variety of different types of neoplasms.

Advances in sequencing technologies and bioinformatics, coupled with the identification of the sequence of the human genome, have enabled more than a dozen tumor types to be evaluated for mutations over their entire exomes [Meyerson et al., 2010; Stratton, 2011]. These studies have demonstrated that the landscape of each particular tumor type is defined by a small number of genes mutated at a high frequency, called “gene mountains” and a larger number of gene “hills” that are present in a smaller proportion of cases [Wood et al., 2007].

Members of our group recently used next generation sequencing to evaluate the exomes of ovarian clear cell carcinomas (OCCCs) and identified truncating mutations in *ARID1A* (MIM# 603024) in 57% of these tumors [Jones et al., 2010]. Independently, Wiegand et al. [2010] discovered a high prevalence of *ARID1A* mutations in both OCCC (45%) and endometriod carcinoma of the ovary (30%). Combining both studies, two mutations were identified in the same tumor in 30% of the mutated cases, which, taken together with the inactivating nature of the mutations and their remarkable frequency, provided unequivocal evidence that *ARID1A* is a tumor suppressor gene in these two tumor types. In addition, loss of *ARID1A* expression was observed in approximately 20% of uterine carcinomas [Wiegand et al., 2011]. In previous studies, chromosomal translocations involving *ARID1A* were identified in a breast and a lung cancer, though the interpretation of these alterations was challenging [Huang et al., 2007].

The protein encoded by *ARID1A* is a key component of the highly conserved SWI–SNF (switch/sucrose non-fermentable) chromatin remodeling complex that uses adenosine triphosphate (ATP)-dependent helicase activities to allow access of transcriptional activators and repressors to DNA [Wang et al., 2004; Wilson and Roberts, 2011]. The protein therefore appears to be involved in regulating processes including DNA repair, differentiation, and development [Weissman et al., 2009]. Functional studies by Nagl et al. [2007] have demonstrated that the SWI–SNF complex suppresses proliferation. The *ARID1A-*encoded protein, BAF250a, is one of two mutually exclusive ARID1 subunits. BAF250a has a DNA-binding domain that specifically binds to AT-rich DNA sequences and is thought to confer specificity to the complex [Wu et al., 2009].

Passenger mutations are best defined as those that do not confer a selective growth advantage to the cells in which they occur, while driver mutations are those which do confer a growth advantage. It is often difficult to distinguish driver mutations from passenger mutations when the mutations occur at low frequency. One of the best examples of this challenge is provided by *IDH1* mutations. A single mutation of *IDH1*, R132H, was discovered in a whole exomic screen of 11 colorectal cancers (CRCs) [Sjöblom et al., 2006]. This mutation was not identified in more than 200 additional CRC samples and was presumed to be a passenger mutation. However, frequent *IDH1* mutations at the identical residue were found when brain tumors, such as lower grade astrocytomas and oligodendrogliomas were evaluated [Parsons et al., 2008; Yan et al., 2009]. Thus, the *IDH1* mutation in that original CRC in retrospect was undoubtedly a driver.

This example illustrates that once a genetic alteration is identified as a driver in one tumor type, infrequent mutations of the same type in the same gene in other tumors can be more reliably interpreted. Given that, it is now known that *ARID1A* is a bona fide tumor suppressor gene in OCCC, we applied this principle to the evaluation of *ARID1A* mutations in other tumor types. As described below, we studied more than 700 different neoplasms of seven different types using Sanger sequencing to determine the contribution of *ARID1A* alterations to tumorigenesis in general.

Somatic mutations were identified in 43 of the 759 neoplasms studied (6%) (Table 1). Eight neoplasms contained two or three (one case) different mutations, presumably on different alleles, so the total number of mutations was 52. A relatively high frequency of mutations was observed in neoplasms of the colon (10%; 12/119), stomach (10%; 10/100), and pancreas (8%; 10/119). Though only a small number of prostate tumors was available for study, we identified two carcinomas with mutations among the 23 studied. Mutations were observed in three of 125 (2%) medulloblastomas, in four of 114 (4%) breast cancers, and in two of 36 (6%) lung carcinomas (Table 1; Fig. 1). No mutations were observed among 34 glioblastomas or 89 leukemias tested.

**Table 1:** Mutations in the Chromatin Remodeling Gene, *ARID1A*

Sample	Tumor type	Nucleotide (genomic)^b^	Nucleotide (cDNA)^c^	Amino acid (protein)	Mutation type	MSI status
399	Breast	g.chr1:26928914delC	c.1323delC	Fs	Indel	MSS
3814	Breast	g.chr1:26979235G>A	c.6259G>A	p.G2087R	Missense	MSS
5887	Breast	g.chr1:26978695A>T	c.5719A>T	p.I1907F	Missense	MSS
C-122	Breast	g.chr1:26965396C>T	c.2830C>T	p.Q944X	Nonsense	MSS
Co001	Colon	g.chr1:26896495delG	c.1014delG	Fs	Indel	MSI-high
Co001	Colon	g.chr1:26973994delC	c.4689delC	Fs	Indel	MSI-high
Co014	Colon	g.chr1:26970279delA	c.3281delA	Fs	Indel	MSI-high
Co024	Colon	g.chr1:26970342delC	c.3344delC	Fs	Indel	MSI-high
Co024	Colon	g.chr1:26978524delG	c.5548delG	Fs	Indel	MSI-high
Co038	Colon	g.chr1:26973659delC	c.4354delC	Fs	Indel	MSI-high
Co038	Colon	g.chr1:26978524delG	c.5548delG	Fs	Indel	MSI-high
Co083	Colon	g.chr1:26978524delG	c.5548delG	Fs	Indel	MSI-high
Co097	Colon	g.chr1:26978524dupG	c.5548dupG	Fs	Indel	MSI-high
Hx132	Colon	g.chr1:26931798delC	c.1848delC	Fs	Indel	ND
Hx132	Colon	g.chr1:26965600_26965602delAAC	c.2944_2946delAAC	In-frame del	Indel	ND
Hx164	Colon	g.chr1:26930536C>T	c.1657C>T	p.Q553X	Nonsense	MSS
Hx245	Colon	g.chr1:26979204C>A	c.6228C>A	p.Y2076X	Nonsense	MSS
Hx290	Colon	g.chr1:26978814_26978820dupACAGAGC (hom)	c.5838_5844dupACAGAGC	Fs	Indel	MSS
Hx308	Colon	g.chr1:26978810_26978811insAGCACAG	c.5834_5835insAGCACAG	Fs	Indel	ND
Hx326	Colon	g.chr1:26962098_26962099dupTA	c.2467_2468dupTA	Fs	Indel	MSS
G07	Gastric	g.chr1:26896360dupC	c.879dupC	Fs	Indel	MSI-high
G08	Gastric	g.chr1:26896308delG	c.827delG	Fs	Indel	MSI-high
G13	Gastric	g.chr1:26974048_26974049delCA	c.4743_4744delCA	Fs	Indel	MSI-high
G13	Gastric	g.chr1:26978524delG	c.5548delG	Fs	Indel	MSI-high
G13	Gastric	g.chr1:26974277C>T	c.4972C>T	p.R1658W	Missense	MSI-high
G18	Gastric	g.chr1:26978335G>T	c.5359G>T	p.E1787X	Nonsense	MSS
G21	Gastric	g.chr1:26978524delG	c.5548delG	Fs	Indel	MSI-high
G24	Gastric	g.chr1:26973829T>A	c.4524T>A	p.Y1508X	Nonsense	MSI-high
G61	Gastric	g.chr1:26978524delG	c.5548delG	Fs	Indel	ND
G61	Gastric	g.chr1:26979396delC	c.6420delC	Fs	Indel	ND
G84	Gastric	g.chr1:26961335dupG	c.2357dupG	Fs	Indel	MSS
G144	Gastric	g.chr1:26896335delG	c.854delG	Fs	Indel	MSS
G280	Gastric	g.chr1:26896450_26896456delGGGCGCC	c.969_975delGGGCGCC	Fs	Indel	MSS
L11C	Lung	g.chr1:26965400delG	c.2834delG	Fs	Indel	ND
L17C	Lung	g.chr1:26979379_26979384delATTCTG	c.6403_6408delATTCTG	In-frame del	Indel	MSS
MB118PT^a^	Medulloblastoma	g.chr1:26896496delG	c.1015delG	Fs	Indel	MSS
MB155PT	Medulloblastoma	g.chr1:26974198_26974199InsC	c.4893_4894InsC	Fs	Indel	MSS
MB156PT	Medulloblastoma	g.chr1:26974673delG	c.5012delG	Fs	Indel	MSS
Pa07C^a^	Pancreas	g.chr1:26972534C>T	c.3826C>T	p.R1276X	Nonsense	MSS
Pa37X	Pancreas	g.chr1:26978923_26978924delTG	c.5947_5948delTG	Fs	Indel	MSS
Pa102C^a^	Pancreas	g.chr1:26965645G>A	IVS10+1G>A	Splice site	Splice site	MSS
Pa144X	Pancreas	g.chr1:26959958_26959959insT	c.1945_1946insT	Fs	Indel	MSS
Pa158X	Pancreas	g.chr1:26961274dupC	c.2296dupC	Fs	Indel	MSS
Pa166X	Pancreas	g.chr1:26978941C>T	c.5965C>T	p.R1989X	Nonsense	MSS
Pa194X	Pancreas	g.chr1:26978941C>T	c.5965C>T	p.R1989X	Nonsense	MSS
Pa194X	Pancreas	g.chr1:26979263C>G	c.6287C>G	p.S2096X	Nonsense	MSS
Pa197X	Pancreas	g.chr1:26930464C>T	c.1585C>T	p.Q529X	Nonsense	MSS
Pa198X	Pancreas	g.chr1:26978524dupG	c.5548dupG	Fs	Indel	MSS
Pa216X	Pancreas	g.chr1:26961380delG	c.2402delG	Fs	Indel	MSS
SW32	Prostate	g.chr1:26972768delC	c.3977delC	Fs	Indel	ND
SW32	Prostate	g.chr1:26978524dupG	c.5548dupG	Fs	Indel	ND
Pr04PT	Prostate	g.chr1:26972790_26972792het_delGCA	c.3999_4101delGCA	In-frame del	Indel	MSI-high

a Mutation previously reported.

b Genomic co-ordinates refer to hg18.

c Reference sequence CCDS285.1.

MSI, microsatellite instability; MSS, microsatellite stable; ND, not determined.

**Figure 1:**
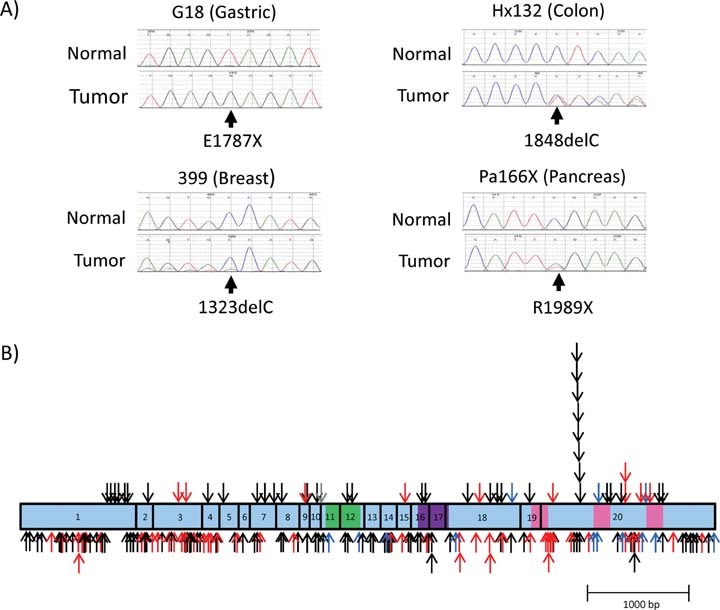
A: Examples of truncating mutations in *ARID1A* in gastric, colon, breast, and pancreatic cancers. Arrows indicate the position of the mutation. Note that in the breast primary tumor (399), there were contaminating nonneoplastic cells that reduced the relative peak heights of the mutant alleles. B: Distribution and types of mutations identified in *ARID1A* to date. Exons are indicated in blue with the ARID (AT-rich interactive domain), DNA-binding domain shown in green, the HIC (hypermethylated in cancer) domain in purple, and the LXXLL (leucine rich) motifs in pink. Black arrows indicate the position of insertion or deletion mutations, red arrows indicate nonsense mutations, blue arrows indicate missense variants, and gray arrows indicate splice site alterations. Mutations listed above the figure represent those reported in this study; those below were identified in Jones et al. [2010] and Wiegand et al. [2010] in ovarian cancers; Gui et al. [2011] in bladder cancer; Varela et al. in renal cancer, and Birnbaum et al. in pancreatic cancer.

As expected for inactivating mutations of a tumor suppressor gene, the mutations were distributed throughout the gene and included nonsense variants, out-of-frame and in-frame small insertions and deletions, as well as a small number (three) of missense changes. Mutations were most commonly observed in a seven-base G tract around position g.chr1:26978524 (genomic coordinates refer to hg18) (c.5548), where there were six single base pair deletions and three duplications among gastric, colon, prostate, and pancreas carcinomas. This G tract is the longest mononucleotide repeat in the coding region and the probability of slippage at mononucleotide repeats clearly increases with run length [Eshleman et al., 1996; Markowitz et al., 1995]. Thirty-eight of the 43 samples with somatic mutations were available for microsatellite instability (MSI) testing. Twelve tumors (six colon, five gastric, and one prostate) were shown to be MSI high, and all carried mutations at mononucleotide tracts in the *ARID1A* gene (Table 1). It is therefore possible that *ARID1A*, such as *TGFβRII* or *BAX*, is associated with MSI and that the homopolymeric repeat frameshifts may result from defects in mismatch repair [Markowitz et al., 1995; Rampino et al., 1997]. Though the interpretation of mutations in mismatch repair-deficient tumors is challenging [Kern, 2002], the fact that approximately 40% of the CRCs with *ARID1A* mutations did not have MSI leaves little doubt that *ARID1A* plays a role in this tumor type.

The identification of mutations in *ARID1A* in several different types of cancer indicates that this gene has a wider role in human tumorigenesis than previously appreciated. These findings are supported by the demonstration of loss of the ARID1A protein, BAF250a, by immunohistochemistry in 14% of gastric and anaplastic thyroid carcinomas [Wiegand et al., 2011] and by the identification of *ARID1A* point mutations in 3 of 48 pancreatic cancers by Birnbaum et al. [2011]. More recently, ARID1A mutations have also been observed in 13% of bladder carcinomas [Gui et al., 2011]. In addition, *ARID1A* appears to be frequently mutated in gastrointestinal tumors displaying high levels of MSI. Mutations in other members of the SWI–SNF chromatin remodeling complex have also been reported. For example, truncating mutations in *SMARCA4/BRG1* were identified in three pancreatic cancers, in a medulloblastoma, and in several lung cancers [Jones et al., 2008; Medina et al., 2008; Parsons et al., 2011]. More recently, 41% of renal cancers have been shown to have truncating mutations in the SWI–SNF chromatin remodeling complex gene, *PBRM1* [Varela et al., 2011]. In addition, a pattern of somatic mutation of genes involved more generally in chromatin remodeling is starting to appear. *MLL3* appears to be involved in a small number of colon and pancreatic cancers and medulloblastomas [Jones et al., 2008; Parsons et al., 2011; Wood et al., 2007]; *MLL2* is mutated in 14% of medulloblastomas and a large fraction of non-Hodgkin's lymphomas [Morin et al., 2011; Parsons et al., 2011] and *JARID1C* is genetically altered in a small proportion of kidney cancers [Dalgliesh et al., 2010]. These data collectively link genetic alterations to epigenetic changes and pave the way for a better understanding of both.
