# Supplementary material for: Somatic Mutations in the Chromatin Remodeling Gene ARID1A Occur in Several Tumor Types
Source: Hum Mutat. 2011 Oct 18;33(1):100–3. doi: 10.1002/humu.21633 (PMC3240719; doi:10.1002/humu.21633)
Supplement: Supplementary file 1 [file humu0033-0100-SD1.pdf]

<u>Supp. Materials and Methods</u>

Samples

A total of 763 neoplasms subdivided into 119 pancreas (2 mutations among 24 samples were previously reported (Jones et al, 2008), 114 breast (0 mutations among 11 samples previously studied (Wood et al, 2007), 36 lung, 100 gastric, 34 glioblastoma (0 mutations among 22 samples previously studied) (Parsons et al, 2008), 125 medulloblastoma (1 mutation among 110 samples was previously reported) (Parsons et al, 2011), 119 colon, 23 prostate and 89 leukemias (11 acute lymphocytic leukemia, 47 acute myelocytic leukemia, 24 chronic lymphocytic leukemia and 7 chronic myelogenous leukemia) were obtained according to appropriate IRB protocols. Tumor DNA was extracted as previously described (Sjoblom et al, 2006). Of the 763 neoplasms, 103 were cell lines and the remainder were primary tumors or xenografts. As we considered only truncating mutations as drivers, and because truncating mutations of *ARID1A* have never been observed in the human germline (Jones et al, 2010; Wiegand et al, 2010; dbSNP (http://www.ncbi.nlm.nih.gov/projects/SNP); 1000 Genomes Consortium, 2010), we considered any truncating mutations to be somatic in origin. The somatic nature of the truncating mutations was confirmed in 100% of the cases in which matched normal DNA was available (21 mutations in 17 tumors).

Amplification

The coding regions of *ARID1A* (CCDS285.1; NM_006015.4; MIM# 603024) were amplified by the polymerase chain reaction in 5 µl reactions containing 1× PCR Buffer (67 mM Tris-HCl, pH 8.8, 6.7 mM $MgCl_2$, 16.6 mM NH_4SO_4, 10 mM 2-mercaptoethanol), 1 mM dNTPs (Invitrogen, San Diego, CA), 1 µM forward and 1 µM reverse primers, 6% DMSO, 2 mM ATP, 0.25 U Platinum Taq (Invitrogen, San Diego, CA) and 3 ng DNA. The 34 pairs of primer sequences used were reported in Jones et al, 2010. PCR cycling conditions were as follows: 94°C for 2 min; three cycles of 94°C for 15 s, 64°C for 30 s, 70°C for 30 s; three cycles of 94°C for 15 s, 61°C for 30 s, 70°C for 30 s; three cycles of 94°C for 15 s, 58°C for 30 s, 70°C for 30 s; and 41 cycles of 94°C for 15 s, 57°C for 30 s, 70°C for 30 s, followed by 70°C for 5 min.

Sequencing

Sequencing was carried out as described in Sjoblom et al. In brief, PCR products were purified using AMPure (Agencourt Biosciences, Beverly, MA) and sequencing was carried out with Big Dye Terminator Kit v.3.1 (Applied Biosystems, Foster City, CA). One PCR primer of each pair was tagged with an M13F sequence (5'-GTAAAACGACGGCCAGT) to allow Sanger sequencing with this universal primer. Sequencing reactions were purified using the CleanSEQ kit (Agencourt Biosciences, Beverly, MA) and run on ABI PRISM 3730 machines (Applied Biosystems, Foster City, CA). Mutation surveyor software (SoftGenetics, State College, PA) was used to visually analyze sequencing traces for mutations and all potential variants were confirmed by an independent PCR and sequencing reaction. Nucleotide numbering reflects cDNA numbering with +1 corresponding to the A of the ATG translation initiation codon in the

reference sequence, according to journal guidelines (www.hgvs.org/mutnomen). The initiation codon is codon 1.

Microsatellite instability testing

Microsatellite instability was detected using the MSI Analysis System (Promega, Madison, WI), which contains 5 mononucleotide repeats (BAT-25, BAT-26, NR-21, NR-24 and MONO-27) and 2 pentanucleotide repeat loci, per manufacturer's instructions. Following amplification, the fluorescent PCR products were sized on an Applied Biosystems 3130 capillary electrophoresis instrument (Invitrogen, Calsbad, CA). Tumor samples were designated as: MSI-high if two or more mononucleotides varied in length compared to the germline DNA, MSI-low if only one locus varied, and microsatellite stable (MSS) if there was no variation compared to the germline. Pentanucleotide loci confirmed identity in all cases where normal DNA was available. For samples lacking normal DNA, tumor microsatellite length was interpreted relative to population length of these generally monomorphic alleles.

References

1000 Genomes Project Consortium. 2010. A map of human genome variation from population-scale sequencing. Nature 467:1061-73.

Jones S, Zhang X, Parsons DW, Lin JC, Leary RJ, Angenendt P, Mankoo P, Carter H, Kamiyama H, Jimeno A, Hong SM, Fu B, Lin MT, Calhoun ES, Kamiyama M, Walter K, Nikolskaya T, Nikolsky Y, Hartigan J, Smith DR, Hidalgo M, Leach SD, Klein AP, Jaffee EM, Goggins M, Maitra A, Iacobuzio-Donahue C, Eshleman JR, Kern SE, Hruban RH, Karchin R, Papadopoulos N, Parmigiani G, Vogelstein B, Velculescu VE, Kinzler KW. 2008. Core signaling pathways in human pancreatic cancers revealed by global genomic analyses. Science 321:1801-6.

Jones S, Wang TL, Shih IeM, Mao TL, Nakayama K, Roden R, Glas R, Slamon D, Diaz LA Jr, Vogelstein B, Kinzler KW, Velculescu VE, Papadopoulos N. 2010. Frequent mutations of chromatin remodeling gene ARID1A in ovarian clear cell carcinoma. Science 330:228-31.

Parsons DW, Jones S, Zhang X, Lin JC, Leary RJ, Angenendt P, Mankoo P, Carter H, Siu IM, Gallia GL, Olivi A, McLendon R, Rasheed BA, Keir S, Nikolskaya T, Nikolsky Y, Busam DA, Tekleab H, Diaz LA Jr, Hartigan J, Smith DR, Strausberg RL,Marie SK, Shinjo SM, Yan H, Riggins GJ, Bigner DD, Karchin R, Papadopoulos N,Parmigiani G, Vogelstein B, Velculescu VE, Kinzler KW. 2008. An integrated genomic analysis of human glioblastoma multiforme. Science 321:1807-12.

Parsons DW, Li M, Zhang X, Jones S, Leary RJ, Lin JC, Boca SM, Carter H, Samayoa J, Bettegowda C, Gallia GL, Jallo GI, Binder ZA, Nikolsky Y, Hartigan J, Smith DR, Gerhard DS, Fults DW, VandenBerg S, Berger MS, Marie SK, Shinjo SM,Clara C, Phillips PC, Minturn JE, Biegel JA, Judkins AR, Resnick AC, Storm PB,Curran T, He Y, Rasheed BA, Friedman HS, Keir ST, McLendon R, Northcott PA,Taylor MD, Burger PC, Riggins GJ, Karchin R, Parmigiani G, Bigner DD, Yan H,Papadopoulos N, Vogelstein B, Kinzler KW, Velculescu VE. 2011. The genetic landscape of the childhood cancer medulloblastoma. Science 331:435-9.

Sjöblom T, Jones S, Wood LD, Parsons DW, Lin J, Barber TD, Mandelker D, Leary RJ, Ptak J, Silliman N, Szabo S, Buckhaults P, Farrell C, Meeh P, Markowitz SD, Willis J, Dawson D, Willson JK, Gazdar AF, Hartigan J, Wu L, Liu C, Parmigiani G, Park BH, Bachman KE, Papadopoulos N, Vogelstein B, Kinzler KW, Velculescu VE. 2006. The consensus coding sequences of human breast and colorectal cancers. Science 314:268-74.

Wiegand KC, Shah SP, Al-Agha OM, Zhao Y, Tse K, Zeng T, Senz J, McConechy MK, Anglesio MS, Kalloger SE, Yang W, Heravi-Moussavi A, Giuliany R, Chow C, Fee J,Zayed A, Prentice L, Melnyk N, Turashvili G, Delaney AD, Madore J, Yip S, McPherson AW, Ha G, Bell L, Fereday S, Tam A, Galletta L, Tonin PN, Provencher D, Miller D, Jones SJ, Moore RA, Morin GB, Oloumi A, Boyd N, Aparicio SA, Shih IeM, Mes-Masson AM, Bowtell DD, Hirst M, Gilks B, Marra MA, Huntsman DG. 2010. ARID1A mutations in endometriosis-associated ovarian carcinomas. N Engl J Med 363:1532-43.

Wood LD, Parsons DW, Jones S, Lin J, Sjöblom T, Leary RJ, Shen D, Boca SM, Barber T, Ptak J, Silliman N, Szabo S, Dezso Z, Ustyanksky V, Nikolskaya T, Nikolsky Y, Karchin R, Wilson PA, Kaminker JS, Zhang Z, Croshaw R, Willis J, Dawson D, Shipitsin M, Willson JK, Sukumar S, Polyak K, Park BH, Pethiyagoda CL,Pant PV, Ballinger DG, Sparks AB, Hartigan J, Smith DR, Suh E, Papadopoulos N, Buckhaults P, Markowitz SD, Parmigiani G, Kinzler KW, Velculescu VE, Vogelstein B. 2007. The genomic landscapes of human breast and colorectal cancers. Science 318:1108-13.

Supp. Table S1. Tumor samples studied

Lab #	Cell line name	Tumor origin	Type of sample
D12	NA	Acute Lymphocytic leukemia	Primary Tumor
D26	NA	Acute Lymphocytic leukemia	Primary Tumor
D28	NA	Acute Lymphocytic leukemia	Primary Tumor
D29	NA	Acute Lymphocytic leukemia	Primary Tumor
D31	NA	Acute Lymphocytic leukemia	Primary Tumor
D36	NA	Acute Lymphocytic leukemia	Primary Tumor
D42	NA	Acute Lymphocytic leukemia	Primary Tumor
D43	NA	Acute Lymphocytic leukemia	Primary Tumor
D44	NA	Acute Lymphocytic leukemia	Primary Tumor
D50	NA	Acute Lymphocytic leukemia	Primary Tumor
D51	NA	Acute Lymphocytic leukemia	Primary Tumor
L1	NA	Acute Myelocytic Leukemia	Primary Tumor
L2	NA	Acute Myelocytic Leukemia	Primary Tumor
L5	NA	Acute Myelocytic Leukemia	Primary Tumor
L13	NA	Acute Myelocytic Leukemia	Primary Tumor
L14	NA	Acute Myelocytic Leukemia	Primary Tumor
L15	NA	Acute Myelocytic Leukemia	Primary Tumor
L18	NA	Acute Myelocytic Leukemia	Primary Tumor
L31	NA	Acute Myelocytic Leukemia	Primary Tumor
L34	NA	Acute Myelocytic Leukemia	Primary Tumor
L39	NA	Acute Myelocytic Leukemia	Primary Tumor
L42	NA	Acute Myelocytic Leukemia	Primary Tumor
L51	NA	Acute Myelocytic Leukemia	Primary Tumor
L52	NA	Acute Myelocytic Leukemia	Primary Tumor
L55	NA	Acute Myelocytic Leukemia	Primary Tumor
L62	NA	Acute Myelocytic Leukemia	Primary Tumor
L64	NA	Acute Myelocytic Leukemia	Primary Tumor
L73	NA	Acute Myelocytic Leukemia	Primary Tumor
L74	NA	Acute Myelocytic Leukemia	Primary Tumor
L75	NA	Acute Myelocytic Leukemia	Primary Tumor
L82	NA	Acute Myelocytic Leukemia	Primary Tumor
L90	NA	Acute Myelocytic Leukemia	Primary Tumor
L96	NA	Acute Myelocytic Leukemia	Primary Tumor
L99	NA	Acute Myelocytic Leukemia	Primary Tumor
L104	NA	Acute Myelocytic Leukemia	Primary Tumor
L120	NA	Acute Myelocytic Leukemia	Primary Tumor
L134	NA	Acute Myelocytic Leukemia	Primary Tumor
L144	NA	Acute Myelocytic Leukemia	Primary Tumor

L175	NA	Acute Myelocytic Leukemia	Primary Tumor
L176	NA	Acute Myelocytic Leukemia	Primary Tumor
L179	NA	Acute Myelocytic Leukemia	Primary Tumor
L220	NA	Acute Myelocytic Leukemia	Primary Tumor
L221	NA	Acute Myelocytic Leukemia	Primary Tumor
L222	NA	Acute Myelocytic Leukemia	Primary Tumor
L223	NA	Acute Myelocytic Leukemia	Primary Tumor
L224	NA	Acute Myelocytic Leukemia	Primary Tumor
L225	NA	Acute Myelocytic Leukemia	Primary Tumor
L226	NA	Acute Myelocytic Leukemia	Primary Tumor
L227	NA	Acute Myelocytic Leukemia	Primary Tumor
L228	NA	Acute Myelocytic Leukemia	Primary Tumor
L232	NA	Acute Myelocytic Leukemia	Primary Tumor
L233	NA	Acute Myelocytic Leukemia	Primary Tumor
L234	NA	Acute Myelocytic Leukemia	Primary Tumor
L235	NA	Acute Myelocytic Leukemia	Primary Tumor
L236	NA	Acute Myelocytic Leukemia	Primary Tumor
L237	NA	Acute Myelocytic Leukemia	Primary Tumor
L238	NA	Acute Myelocytic Leukemia	Primary Tumor
L239	NA	Acute Myelocytic Leukemia	Primary Tumor
C-19	2R-75-30	Breast	Cell line
C-30	BT123	Breast	Cell line
C-33	MDA MB 157	Breast	Cell line
C-34	MDA MB 231	Breast	Cell line
C-44	TT	Breast	Cell line
C-60	MCF7	Breast	Cell line
C-122	T47D	Breast	Cell line
201	NA	Breast	Primary tumor
250	NA	Breast	Primary tumor
273	NA	Breast	Primary tumor
317	NA	Breast	Primary tumor
333	NA	Breast	Primary tumor
399	NA	Breast	Primary tumor
409	NA	Breast	Primary tumor
418	NA	Breast	Primary tumor
423	NA	Breast	Primary tumor
428	NA	Breast	Primary tumor
565	NA	Breast	Primary tumor
699	NA	Breast	Primary tumor
701	NA	Breast	Primary tumor
805	NA	Breast	Primary tumor
829	NA	Breast	Primary tumor
914	NA	Breast	Primary tumor

953	NA	Breast	Primary tumor
969	NA	Breast	Primary tumor
1214	NA	Breast	Primary tumor
1250	NA	Breast	Primary tumor
1267	NA	Breast	Primary tumor
1386	NA	Breast	Primary tumor
1401	NA	Breast	Primary tumor
1413	NA	Breast	Primary tumor
1587	NA	Breast	Primary tumor
1613	NA	Breast	Primary tumor
1694	NA	Breast	Primary tumor
1717	NA	Breast	Primary tumor
1759	NA	Breast	Primary tumor
1784	NA	Breast	Primary tumor
1855	NA	Breast	Primary tumor
1913	NA	Breast	Primary tumor
1957	NA	Breast	Primary tumor
2201	NA	Breast	Primary tumor
2338	NA	Breast	Primary tumor
2425	NA	Breast	Primary tumor
2647	NA	Breast	Primary tumor
2659	NA	Breast	Primary tumor
2911	NA	Breast	Primary tumor
3008	NA	Breast	Primary tumor
3200	NA	Breast	Primary tumor
3382	NA	Breast	Primary tumor
3474	NA	Breast	Primary tumor
3539	NA	Breast	Primary tumor
3568	NA	Breast	Primary tumor
3594	NA	Breast	Primary tumor
3809	NA	Breast	Primary tumor
3814	NA	Breast	Primary tumor
3904	NA	Breast	Primary tumor
4023	NA	Breast	Primary tumor
4061	NA	Breast	Primary tumor
4171	NA	Breast	Primary tumor
4274	NA	Breast	Primary tumor
4380	NA	Breast	Primary tumor
4444	NA	Breast	Primary tumor
4541	NA	Breast	Primary tumor
4554	NA	Breast	Primary tumor
4573	NA	Breast	Primary tumor
4581	NA	Breast	Primary tumor

4691	NA	Breast	Primary tumor
4756	NA	Breast	Primary tumor
5015	NA	Breast	Primary tumor
5038	NA	Breast	Primary tumor
5125	NA	Breast	Primary tumor
5142	NA	Breast	Primary tumor
5281	NA	Breast	Primary tumor
5287	NA	Breast	Primary tumor
5297	NA	Breast	Primary tumor
5322	NA	Breast	Primary tumor
5327	NA	Breast	Primary tumor
5355	NA	Breast	Primary tumor
5422	NA	Breast	Primary tumor
5500	NA	Breast	Primary tumor
5508	NA	Breast	Primary tumor
5565	NA	Breast	Primary tumor
5646	NA	Breast	Primary tumor
5690	NA	Breast	Primary tumor
5761	NA	Breast	Primary tumor
5887	NA	Breast	Primary tumor
6271	NA	Breast	Primary tumor
6380	NA	Breast	Primary tumor
6407	NA	Breast	Primary tumor
6409	NA	Breast	Primary tumor
6566	NA	Breast	Primary tumor
6704	NA	Breast	Primary tumor
6724	NA	Breast	Primary tumor
6727	NA	Breast	Primary tumor
6785	NA	Breast	Primary tumor
6799	NA	Breast	Primary tumor
6810	NA	Breast	Primary tumor
6864	NA	Breast	Primary tumor
7105	NA	Breast	Primary tumor
7122	NA	Breast	Primary tumor
7152	NA	Breast	Primary tumor
7154	NA	Breast	Primary tumor
7157	NA	Breast	Primary tumor
HCC38	HCC38	Breast	Cell line
HCC1008	HCC1008	Breast	Cell line
HCC1143	HCC1143	Breast	Cell line
HCC1187	HCC1187	Breast	Cell line
HCC1395	HCC1395	Breast	Cell line
HCC1599	HCC1599	Breast	Cell line

HCC1937	HCC1937	Breast	Cell line
HCC1954	HCC1954	Breast	Cell line
HCC2157	HCC2157	Breast	Cell line
HCC2218	HCC2218	Breast	Cell line
Hs578T	Hs578T	Breast	Cell line
CLL-1	NA	Chronic Lymphocytic Leukemia	Primary tumor
CLL-5	NA	Chronic Lymphocytic Leukemia	Primary tumor
CLL-6	NA	Chronic Lymphocytic Leukemia	Primary tumor
CLL-10	NA	Chronic Lymphocytic Leukemia	Primary tumor
CLL-11	NA	Chronic Lymphocytic Leukemia	Primary tumor
CLL-20	NA	Chronic Lymphocytic Leukemia	Primary tumor
CLL-27	NA	Chronic Lymphocytic Leukemia	Primary tumor
CLL-29	NA	Chronic Lymphocytic Leukemia	Primary tumor
CLL-43	NA	Chronic Lymphocytic Leukemia	Primary tumor
CLL-50	NA	Chronic Lymphocytic Leukemia	Primary tumor
CLL-52	NA	Chronic Lymphocytic Leukemia	Primary tumor
CLL-55	NA	Chronic Lymphocytic Leukemia	Primary tumor
CLL-58	NA	Chronic Lymphocytic Leukemia	Primary tumor
CLL-67	NA	Chronic Lymphocytic Leukemia	Primary tumor
CLL-79	NA	Chronic Lymphocytic Leukemia	Primary tumor
CLL-80	NA	Chronic Lymphocytic Leukemia	Primary tumor
CLL-81	NA	Chronic Lymphocytic Leukemia	Primary tumor
CLL-86	NA	Chronic Lymphocytic Leukemia	Primary tumor
CLL-87	NA	Chronic Lymphocytic Leukemia	Primary tumor
CLL-89	NA	Chronic Lymphocytic Leukemia	Primary tumor
CLL-101	NA	Chronic Lymphocytic Leukemia	Primary tumor
CLL-105	NA	Chronic Lymphocytic Leukemia	Primary tumor
CLL-121	NA	Chronic Lymphocytic Leukemia	Primary tumor
CLL-146	NA	Chronic Lymphocytic Leukemia	Primary tumor
CML-1	NA	Chronic myelogenous leukemia	Primary tumor
CML-3	NA	Chronic myelogenous leukemia	Primary tumor
CML-5	NA	Chronic myelogenous leukemia	Primary tumor
CML-8	NA	Chronic myelogenous leukemia	Primary tumor
CML-16	NA	Chronic myelogenous leukemia	Primary tumor
CML-18	NA	Chronic myelogenous leukemia	Primary tumor
CML-20	NA	Chronic myelogenous leukemia	Primary tumor
Co001	SW 48	Colon	Cell line
Co002	SW 403	Colon	Cell line
Co004	SW 837	Colon	Cell line
Co005	SW 948	Colon	Cell line
Co006	SW 1116	Colon	Cell line
Co007	SW 1417	Colon	Cell line
Co008	SW 1463	Colon	Cell line

Co010	LOVO	Colon	Cell line
Co011	COLO 205	Colon	Cell line
Co012	SK-CO-1	Colon	Cell line
Co013	HT29	Colon	Cell line
Co014	LS180	Colon	Cell line
Co020	DLD-1	Colon	Cell line
Co024	RKO	Colon	Cell line
Co028	GAZDAR SNU	Colon	Cell line
Co037	SW 480	Colon	Cell line
Co038	HCT 116	Colon	Cell line
Co083	VACO 444	Colon	Cell line
Co096	HC 2998	Colon	Cell line
Co097	KM 12	Colon	Cell line
Cx003	NA	Colon	Xenograft
Cx004	NA	Colon	Xenograft
Cx024	NA	Colon	Xenograft
Hx004	NA	Colon	Xenograft
Hx018	NA	Colon	Xenograft
Hx053	NA	Colon	Xenograft
Hx058	NA	Colon	Xenograft
Hx062	NA	Colon	Xenograft
Hx063	NA	Colon	Xenograft
Hx065	NA	Colon	Xenograft
Hx067	NA	Colon	Xenograft
Hx080	NA	Colon	Xenograft
Hx083	NA	Colon	Xenograft
Hx089	NA	Colon	Xenograft
Hx094	NA	Colon	Xenograft
Hx098	NA	Colon	Xenograft
Hx100	NA	Colon	Xenograft
Hx104	NA	Colon	Xenograft
Hx108	NA	Colon	Xenograft
Hx110	NA	Colon	Xenograft
Hx130	NA	Colon	Xenograft
Hx132	NA	Colon	Xenograft
Hx133	NA	Colon	Xenograft
Hx134	NA	Colon	Xenograft
Hx135	NA	Colon	Xenograft
Hx139	NA	Colon	Xenograft
Hx140	NA	Colon	Xenograft
Hx147	NA	Colon	Xenograft
Hx149	NA	Colon	Xenograft
Hx152	NA	Colon	Xenograft

Hx164	NA	Colon	Xenograft
Hx166	NA	Colon	Xenograft
Hx168	NA	Colon	Xenograft
Hx170	NA	Colon	Xenograft
Hx175	NA	Colon	Xenograft
Hx178	NA	Colon	Xenograft
Hx181	NA	Colon	Xenograft
Hx186	NA	Colon	Xenograft
Hx192	NA	Colon	Xenograft
Hx196	NA	Colon	Xenograft
Hx198	NA	Colon	Xenograft
Hx199	NA	Colon	Xenograft
Hx205	NA	Colon	Xenograft
Hx208	NA	Colon	Xenograft
Hx209	NA	Colon	Xenograft
Hx211	NA	Colon	Xenograft
Hx212	NA	Colon	Xenograft
Hx213	NA	Colon	Xenograft
Hx214	NA	Colon	Xenograft
Hx227	NA	Colon	Xenograft
Hx231	NA	Colon	Xenograft
Hx233	NA	Colon	Xenograft
Hx234	NA	Colon	Xenograft
Hx235	NA	Colon	Xenograft
Hx237	NA	Colon	Xenograft
Hx241	NA	Colon	Xenograft
Hx242	NA	Colon	Xenograft
Hx243	NA	Colon	Xenograft
Hx244	NA	Colon	Xenograft
Hx245	NA	Colon	Xenograft
Hx250	NA	Colon	Xenograft
Hx266	NA	Colon	Xenograft
Hx268	NA	Colon	Xenograft
Hx273	NA	Colon	Xenograft
Hx279	NA	Colon	Xenograft
Hx286	NA	Colon	Xenograft
Hx288	NA	Colon	Xenograft
Hx290	NA	Colon	Xenograft
Hx292	NA	Colon	Xenograft
Hx295	NA	Colon	Xenograft
Hx296	NA	Colon	Xenograft
Hx297	NA	Colon	Xenograft
Hx299	NA	Colon	Xenograft

Hx300	NA	Colon	Xenograft
Hx302	NA	Colon	Xenograft
Hx303	NA	Colon	Xenograft
Hx305	NA	Colon	Xenograft
Hx306	NA	Colon	Xenograft
Hx307	NA	Colon	Xenograft
Hx308	NA	Colon	Xenograft
Hx309	NA	Colon	Xenograft
Hx310	NA	Colon	Xenograft
Hx312	NA	Colon	Xenograft
Hx313	NA	Colon	Xenograft
Hx314	NA	Colon	Xenograft
Hx315	NA	Colon	Xenograft
Hx317	NA	Colon	Xenograft
Hx318	NA	Colon	Xenograft
Hx319	NA	Colon	Xenograft
Hx321	NA	Colon	Xenograft
Hx322	NA	Colon	Xenograft
Hx324	NA	Colon	Xenograft
Hx325	NA	Colon	Xenograft
Hx326	NA	Colon	Xenograft
Hx327	NA	Colon	Xenograft
Mx001	NA	Colon	Xenograft
Mx021	NA	Colon	Xenograft
Mx028	NA	Colon	Xenograft
Mx039	NA	Colon	Xenograft
G01	NA	Gastric	Xenograft
G02	NA	Gastric	Xenograft
G03	NA	Gastric	Xenograft
G04	NA	Gastric	Xenograft
G05	NA	Gastric	Xenograft
G06	NA	Gastric	Xenograft
G07	NA	Gastric	Xenograft
G08	NA	Gastric	Xenograft
G09	NA	Gastric	Xenograft
G10	NA	Gastric	Xenograft
G11	NA	Gastric	Xenograft
G12	NA	Gastric	Xenograft
G13	NA	Gastric	Xenograft
G14	NA	Gastric	Xenograft
G15	NA	Gastric	Xenograft
G16	NA	Gastric	Xenograft
G17	NA	Gastric	Xenograft

G18	NA	Gastric	Xenograft
G19	NA	Gastric	Xenograft
G20	NA	Gastric	Xenograft
G21	NA	Gastric	Xenograft
G22	NA	Gastric	Xenograft
G23	NA	Gastric	Xenograft
G24	NA	Gastric	Xenograft
G25	NA	Gastric	Xenograft
G26	NA	Gastric	Xenograft
G27	NA	Gastric	Xenograft
G28	NA	Gastric	Xenograft
G29	NA	Gastric	Xenograft
G31	NA	Gastric	Xenograft
G32	NA	Gastric	Xenograft
G33	NA	Gastric	Xenograft
G34	NA	Gastric	Xenograft
G35	NA	Gastric	Xenograft
G36	NA	Gastric	Xenograft
G39	NA	Gastric	Xenograft
G40	NA	Gastric	Xenograft
G41	NA	Gastric	Xenograft
G42	NA	Gastric	Xenograft
G43	NA	Gastric	Xenograft
G44	NA	Gastric	Xenograft
G45	NA	Gastric	Xenograft
G46	NA	Gastric	Xenograft
G47	NA	Gastric	Xenograft
G48	NA	Gastric	Xenograft
G49	NA	Gastric	Xenograft
G50	NA	Gastric	Xenograft
G51	NA	Gastric	Xenograft
G52	NA	Gastric	Xenograft
G53	NA	Gastric	Xenograft
G54	NA	Gastric	Xenograft
G55	NA	Gastric	Xenograft
G59	NA	Gastric	Xenograft
G63	NA	Gastric	Xenograft
G64	NA	Gastric	Xenograft
G65	NA	Gastric	Xenograft
G108PT	NA	Gastric	Primary tumor
G110PT	NA	Gastric	Primary tumor
G113PT	NA	Gastric	Primary tumor
G115PT	NA	Gastric	Primary tumor

G118PT	NA	Gastric	Primary tumor
G119PT	NA	Gastric	Primary tumor
G123PT	NA	Gastric	Primary tumor
G125PT	NA	Gastric	Primary tumor
G1275PT	NA	Gastric	Primary tumor
G1296PT	NA	Gastric	Primary tumor
G132PT	NA	Gastric	Primary tumor
G144PT	NA	Gastric	Primary tumor
G1546PT	NA	Gastric	Primary tumor
G212PT	NA	Gastric	Primary tumor
G273PT	NA	Gastric	Primary tumor
G280PT	NA	Gastric	Primary tumor
G295PT	NA	Gastric	Primary tumor
G296PT	NA	Gastric	Primary tumor
G332PT	NA	Gastric	Primary tumor
G401PT	NA	Gastric	Primary tumor
G402PT	NA	Gastric	Primary tumor
G403PT	NA	Gastric	Primary tumor
G404PT	NA	Gastric	Primary tumor
G406PT	NA	Gastric	Primary tumor
G407PT	NA	Gastric	Primary tumor
G408PT	NA	Gastric	Primary tumor
G409PT	NA	Gastric	Primary tumor
G410PT	NA	Gastric	Primary tumor
G59PT	NA	Gastric	Primary tumor
G61PT	NA	Gastric	Primary tumor
G62PT	NA	Gastric	Primary tumor
G72PT	NA	Gastric	Primary tumor
G79PT	NA	Gastric	Primary tumor
G801PT	NA	Gastric	Primary tumor
G802PT	NA	Gastric	Primary tumor
G803PT	NA	Gastric	Primary tumor
G804PT	NA	Gastric	Primary tumor
G805PT	NA	Gastric	Primary tumor
G806PT	NA	Gastric	Primary tumor
G807PT	NA	Gastric	Primary tumor
G808PT	NA	Gastric	Primary tumor
G82PT	NA	Gastric	Primary tumor
G84PT	NA	Gastric	Primary tumor
G88PT	NA	Gastric	Primary tumor
GLI-01X	NA	Glioblastoma	Xenograft
GLI-02X	NA	Glioblastoma	Xenograft
GLI-03X	NA	Glioblastoma	Xenograft

GLI-04X	NA	Glioblastoma	Xenograft
GLI-05X	NA	Glioblastoma	Xenograft
GLI-06X	NA	Glioblastoma	Xenograft
GLI-07X	NA	Glioblastoma	Xenograft
GLI-08X	NA	Glioblastoma	Xenograft
GLI-09X	NA	Glioblastoma	Xenograft
GLI-10X	NA	Glioblastoma	Xenograft
GB15T	NA	Glioblastoma	Primary Tumor
GB16C	SF188	Glioblastoma	Cell line
Br02X	NA	Glioblastoma	Xenograft
Br03X	NA	Glioblastoma	Xenograft
Br04X	NA	Glioblastoma	Xenograft
Br05X	NA	Glioblastoma	Xenograft
Br06X	NA	Glioblastoma	Xenograft
Br07X	NA	Glioblastoma	Xenograft
Br08X	NA	Glioblastoma	Xenograft
Br09P	NA	Glioblastoma	Primary Tumor
Br10P	NA	Glioblastoma	Primary Tumor
Br11P	NA	Glioblastoma	Primary Tumor
Br12P	NA	Glioblastoma	Primary Tumor
Br13X	NA	Glioblastoma	Xenograft
Br14X	NA	Glioblastoma	Xenograft
Br15X	NA	Glioblastoma	Xenograft
Br16X	NA	Glioblastoma	Xenograft
Br17X	NA	Glioblastoma	Xenograft
Br20P	NA	Glioblastoma	Primary Tumor
Br23X	NA	Glioblastoma	Xenograft
Br25X	NA	Glioblastoma	Xenograft
Br26X	NA	Glioblastoma	Xenograft
Br27P	NA	Glioblastoma	Primary Tumor
Br29P	NA	Glioblastoma	Primary Tumor
C-51	CaluI	Lung	Cell line
L01C	H1395	Lung	Cell line
L02C	H1437	Lung	Cell line
L03C	H2009	Lung	Cell line
L04C	H2122	Lung	Cell line
L05C	H2087	Lung	Cell line
L06C	H2171	Lung	Cell line
L07C	H2195	Lung	Cell line
L08C	H1184	Lung	Cell line
L09C	H209	Lung	Cell line
L10C	H2107	Lung	Cell line
L11C	H128	Lung	Cell line

L12C	HCC193	Lung	Cell line
L13C	HCC15	Lung	Cell line
L14C	HCC78	Lung	Cell line
L15C	HCC366	Lung	Cell line
L16C	HCC515	Lung	Cell line
L17C	HCC20	Lung	Cell line
L18C	HCC2429	Lung	Cell line
L19C	H1450	Lung	Cell line
L20C	HCC33	Lung	Cell line
L21C	H970	Lung	Cell line
L22C	H1607	Lung	Cell line
L23C	H2028	Lung	Cell line
L24C	HCC95	Lung	Cell line
L25C	H2141	Lung	Cell line
L26C	HCC827	Lung	Cell line
L27C	H1339	Lung	Cell line
L28C	H1672	Lung	Cell line
L29C	H1963	Lung	Cell line
L30C	H2052	Lung	Cell line
L31C	H2887	Lung	Cell line
L32C	H2882	Lung	Cell line
L33C	H2126	Lung	Cell line
L34C	H2347	Lung	Cell line
L35C	H90	Lung	Cell line
M01T	NA	Medulloblastoma	Primary tumor
M04T	NA	Medulloblastoma	Primary tumor
M05T	NA	Medulloblastoma	Primary tumor
M07T	NA	Medulloblastoma	Primary tumor
M08T	NA	Medulloblastoma	Primary tumor
M09T	NA	Medulloblastoma	Primary tumor
M10T	NA	Medulloblastoma	Primary tumor
M11T	NA	Medulloblastoma	Primary tumor
M12T	NA	Medulloblastoma	Primary tumor
M13X	NA	Medulloblastoma	Xenograft
M14X	NA	Medulloblastoma	Xenograft
M15X	NA	Medulloblastoma	Xenograft
M16X	NA	Medulloblastoma	Xenograft
M17X	NA	Medulloblastoma	Xenograft
M19X	NA	Medulloblastoma	Xenograft
M22X	NA	Medulloblastoma	Xenograft
M25X	NA	Medulloblastoma	Xenograft
M26X	NA	Medulloblastoma	Xenograft
MB101X	NA	Medulloblastoma	Xenograft

MB104X	NA	Medulloblastoma	Xenograft
MB105X	NA	Medulloblastoma	Xenograft
MB106X	NA	Medulloblastoma	Xenograft
MB108C	H721	Medulloblastoma	Cell line
MB109PT	NA	Medulloblastoma	Primary tumor
MB110PT	NA	Medulloblastoma	Primary tumor
MB111PT	NA	Medulloblastoma	Primary tumor
MB112PT	NA	Medulloblastoma	Primary tumor
MB113PT	NA	Medulloblastoma	Primary tumor
MB114PT	NA	Medulloblastoma	Primary tumor
MB115PT	NA	Medulloblastoma	Primary tumor
MB116PT	NA	Medulloblastoma	Primary tumor
MB117PT	NA	Medulloblastoma	Primary tumor
MB118PT	NA	Medulloblastoma	Primary tumor
MB119PT	NA	Medulloblastoma	Primary tumor
MB120PT	NA	Medulloblastoma	Primary tumor
MB121PT	NA	Medulloblastoma	Primary tumor
MB122PT	NA	Medulloblastoma	Primary tumor
MB123PT	NA	Medulloblastoma	Primary tumor
MB124PT	NA	Medulloblastoma	Primary tumor
MB125PT	NA	Medulloblastoma	Primary tumor
MB126PT	NA	Medulloblastoma	Primary tumor
MB127PT	NA	Medulloblastoma	Primary tumor
MB128PT	NA	Medulloblastoma	Primary tumor
MB129PT	NA	Medulloblastoma	Primary tumor
MB130PT	NA	Medulloblastoma	Primary tumor
MB131PT	NA	Medulloblastoma	Primary tumor
MB132PT	NA	Medulloblastoma	Primary tumor
MB133PT	NA	Medulloblastoma	Primary tumor
MB134PT	NA	Medulloblastoma	Primary tumor
MB135PT	NA	Medulloblastoma	Primary tumor
MB140PT	NA	Medulloblastoma	Primary tumor
MB141PT	NA	Medulloblastoma	Primary tumor
MB142PT	NA	Medulloblastoma	Primary tumor
MB143PT	NA	Medulloblastoma	Primary tumor
MB144PT	NA	Medulloblastoma	Primary tumor
MB145PT	NA	Medulloblastoma	Primary tumor
MB146PT	NA	Medulloblastoma	Primary tumor
MB147PT	NA	Medulloblastoma	Primary tumor
MB148PT	NA	Medulloblastoma	Primary tumor
MB149PT	NA	Medulloblastoma	Primary tumor
MB150PT	NA	Medulloblastoma	Primary tumor
MB151PT	NA	Medulloblastoma	Primary tumor

MB152PT	NA	Medulloblastoma	Primary tumor
MB153PT	NA	Medulloblastoma	Primary tumor
MB154PT	NA	Medulloblastoma	Primary tumor
MB155PT	NA	Medulloblastoma	Primary tumor
MB156PT	NA	Medulloblastoma	Primary tumor
MB157PT	NA	Medulloblastoma	Primary tumor
MB158PT	NA	Medulloblastoma	Primary tumor
MB159PT	NA	Medulloblastoma	Primary tumor
MB160PT	NA	Medulloblastoma	Primary tumor
MB161PT	NA	Medulloblastoma	Primary tumor
MB162PT	NA	Medulloblastoma	Primary tumor
MB163PT	NA	Medulloblastoma	Primary tumor
MB164PT	NA	Medulloblastoma	Primary tumor
MB201PT	NA	Medulloblastoma	Primary tumor
MB202PT	NA	Medulloblastoma	Primary tumor
MB203PT	NA	Medulloblastoma	Primary tumor
MB204PT	NA	Medulloblastoma	Primary tumor
MB205PT	NA	Medulloblastoma	Primary tumor
MB206PT	NA	Medulloblastoma	Primary tumor
MB210PT	NA	Medulloblastoma	Primary tumor
MB211PT	NA	Medulloblastoma	Primary tumor
MB212PT	NA	Medulloblastoma	Primary tumor
MB213PT	NA	Medulloblastoma	Primary tumor
MB214PT	NA	Medulloblastoma	Primary tumor
MB215PT	NA	Medulloblastoma	Primary tumor
MB216PT	NA	Medulloblastoma	Primary tumor
MB217PT	NA	Medulloblastoma	Primary tumor
MB218PT	NA	Medulloblastoma	Primary tumor
MB219PT	NA	Medulloblastoma	Primary tumor
MB220PT	NA	Medulloblastoma	Primary tumor
MB221PT	NA	Medulloblastoma	Primary tumor
MB222PT	NA	Medulloblastoma	Primary tumor
MB223PT	NA	Medulloblastoma	Primary tumor
MB224PT	NA	Medulloblastoma	Primary tumor
MB225PT	NA	Medulloblastoma	Primary tumor
MB226PT	NA	Medulloblastoma	Primary tumor
MB227PT	NA	Medulloblastoma	Primary tumor
MB228PT	NA	Medulloblastoma	Primary tumor
MB229PT	NA	Medulloblastoma	Primary tumor
MB230PT	NA	Medulloblastoma	Primary tumor
MB231PT	NA	Medulloblastoma	Primary tumor
MB232PT	NA	Medulloblastoma	Primary tumor
MB233PT	NA	Medulloblastoma	Primary tumor

MB234PT	NA	Medulloblastoma	Primary tumor
MB235PT	NA	Medulloblastoma	Primary tumor
MB236PT	NA	Medulloblastoma	Primary tumor
MB237PT	NA	Medulloblastoma	Primary tumor
MB238PT	NA	Medulloblastoma	Primary tumor
MB239PT	NA	Medulloblastoma	Primary tumor
MB240PT	NA	Medulloblastoma	Primary tumor
MB241PT	NA	Medulloblastoma	Primary tumor
MB242PT	NA	Medulloblastoma	Primary tumor
MB243PT	NA	Medulloblastoma	Primary tumor
MB244PT	NA	Medulloblastoma	Primary tumor
MB245PT	NA	Medulloblastoma	Primary tumor
MB246PT	NA	Medulloblastoma	Primary tumor
MB247PT	NA	Medulloblastoma	Primary tumor
MB248PT	NA	Medulloblastoma	Primary tumor
MB249PT	NA	Medulloblastoma	Primary tumor
MB250PT	NA	Medulloblastoma	Primary tumor
MB251PT	NA	Medulloblastoma	Primary tumor
MB252PT	NA	Medulloblastoma	Primary tumor
MB253PT	NA	Medulloblastoma	Primary tumor
Pa019C	PANC 6.03	Pancreas	Cell line
Pa01C	A2.1	Pancreas	Cell line
Pa022C	TS 0129	Pancreas	Cell line
Pa02C	A6L	Pancreas	Cell line
Pa032X	NA	Pancreas	Xenograft
Pa038X	NA	Pancreas	Xenograft
Pa03C	A10-7	Pancreas	Cell line
Pa044X	NA	Pancreas	Xenograft
Pa04C	JD13D	Pancreas	Cell line
Pa05X	NA	Pancreas	Xenograft
Pa07C	A32-1	Pancreas	Cell line
Pa08C	A38-5	Pancreas	Cell line
Pa09C	PANC 215	Pancreas	Cell line
Pa101C	A61-5	Pancreas	Cell line
Pa102C	PANC 480	Pancreas	Cell line
Pa104C	PANC 2.8	Pancreas	Cell line
Pa105C	PANC 6.03	Pancreas	Cell line
Pa10X	NA	Pancreas	Xenograft
Pa112C	TS0226	Pancreas	Cell line
Pa113C	PANC 2.43	Pancreas	Cell line
Pa116X	NA	Pancreas	Xenograft
Pa118X	NA	Pancreas	Xenograft
Pa119X	NA	Pancreas	Xenograft

Pa11X	NA	Pancreas	Xenograft
Pa120X	NA	Pancreas	Xenograft
Pa121X	NA	Pancreas	Xenograft
Pa122X	NA	Pancreas	Xenograft
Pa124X	NA	Pancreas	Xenograft
Pa125X	NA	Pancreas	Xenograft
Pa126X	NA	Pancreas	Xenograft
Pa127X	NA	Pancreas	Xenograft
Pa131X	NA	Pancreas	Xenograft
Pa132X	NA	Pancreas	Xenograft
Pa133X	NA	Pancreas	Xenograft
Pa135X	NA	Pancreas	Xenograft
Pa136X	NA	Pancreas	Xenograft
Pa137X	NA	Pancreas	Xenograft
Pa138X	NA	Pancreas	Xenograft
Pa139X	NA	Pancreas	Xenograft
Pa140X	NA	Pancreas	Xenograft
Pa141X	NA	Pancreas	Xenograft
Pa144X	NA	Pancreas	Xenograft
Pa145X	NA	Pancreas	Xenograft
Pa146X	NA	Pancreas	Xenograft
Pa147X	NA	Pancreas	Xenograft
Pa148X	NA	Pancreas	Xenograft
Pa149X	NA	Pancreas	Xenograft
Pa14C	PANC 8.13	Pancreas	Cell line
Pa151X	NA	Pancreas	Xenograft
Pa152X	NA	Pancreas	Xenograft
Pa153X	NA	Pancreas	Xenograft
Pa154X	NA	Pancreas	Xenograft
Pa155X	NA	Pancreas	Xenograft
Pa156X	NA	Pancreas	Xenograft
Pa157X	NA	Pancreas	Xenograft
Pa158X	NA	Pancreas	Xenograft
Pa159X	NA	Pancreas	Xenograft
Pa160X	NA	Pancreas	Xenograft
Pa161X	NA	Pancreas	Xenograft
Pa163X	NA	Pancreas	Xenograft
Pa165X	NA	Pancreas	Xenograft
Pa166X	NA	Pancreas	Xenograft
Pa167X	NA	Pancreas	Xenograft
Pa168X	NA	Pancreas	Xenograft
Pa169X	NA	Pancreas	Xenograft
Pa16C	PANC 10.05	Pancreas	Cell line

Pa170X	NA	Pancreas	Xenograft
Pa172X	NA	Pancreas	Xenograft
Pa173X	NA	Pancreas	Xenograft
Pa174X	NA	Pancreas	Xenograft
Pa176X	NA	Pancreas	Xenograft
Pa177X	NA	Pancreas	Xenograft
Pa178X	NA	Pancreas	Xenograft
Pa17C	PANC 4.03	Pancreas	Cell line
Pa181X	NA	Pancreas	Xenograft
Pa182X	NA	Pancreas	Xenograft
Pa183X	NA	Pancreas	Xenograft
Pa184X	NA	Pancreas	Xenograft
Pa185X	NA	Pancreas	Xenograft
Pa187X	NA	Pancreas	Xenograft
Pa189X	NA	Pancreas	Xenograft
Pa18C	PANC 5.04	Pancreas	Cell line
Pa192X	NA	Pancreas	Xenograft
Pa194X	NA	Pancreas	Xenograft
Pa196X	NA	Pancreas	Xenograft
Pa197X	NA	Pancreas	Xenograft
Pa198X	NA	Pancreas	Xenograft
Pa199X	NA	Pancreas	Xenograft
Pa200X	NA	Pancreas	Xenograft
Pa201X	NA	Pancreas	Xenograft
Pa202X	NA	Pancreas	Xenograft
Pa203X	NA	Pancreas	Xenograft
Pa204X	NA	Pancreas	Xenograft
Pa205X	NA	Pancreas	Xenograft
Pa206X	NA	Pancreas	Xenograft
Pa209X	NA	Pancreas	Xenograft
Pa20C	PANC 198	Pancreas	Cell line
Pa210X	NA	Pancreas	Xenograft
Pa211X	NA	Pancreas	Xenograft
Pa212X	NA	Pancreas	Xenograft
Pa214X	NA	Pancreas	Xenograft
Pa216X	NA	Pancreas	Xenograft
Pa217X	NA	Pancreas	Xenograft
Pa218X	NA	Pancreas	Xenograft
Pa21C	PANC 2.5	Pancreas	Cell line
Pa220X	NA	Pancreas	Xenograft
Pa221C	PANC 9.6.94	Pancreas	Cell line
Pa222C	PANC 486	Pancreas	Cell line
Pa223C	PANC 233	Pancreas	Cell line

Pa28C	PANC 3.014	Pancreas	Cell line
Pa29C	TS 0111	Pancreas	Cell line
Pa33X	NA	Pancreas	Xenograft
Pa34X	NA	Pancreas	Xenograft
Pa36X	NA	Pancreas	Xenograft
Pa37X	NA	Pancreas	Xenograft
Pa38X	NA	Pancreas	Xenograft
Pa39X	NA	Pancreas	Xenograft
Pa41X	NA	Pancreas	Xenograft
Pa43X	NA	Pancreas	Xenograft
16S2	NA	Prostate	Primary Tumor
29N4	NA	Prostate	Primary Tumor
31S1	NA	Prostate	Primary Tumor
31S4	NA	Prostate	Primary Tumor
40S1	NA	Prostate	Primary Tumor
98S3	NA	Prostate	Primary Tumor
PR01X	NA	Prostate	Xenograft
PR02X	NA	Prostate	Xenograft
PR04X	NA	Prostate	Xenograft
PR05X	NA	Prostate	Xenograft
PR06X	NA	Prostate	Xenograft
PR07X	NA	Prostate	Xenograft
PR08X	NA	Prostate	Xenograft
PR09X	NA	Prostate	Xenograft
PR11PT	NA	Prostate	Primary tumor
PR12PT	NA	Prostate	Primary tumor
PR14PT	NA	Prostate	Primary tumor
PR15PT	NA	Prostate	Primary tumor
PR16PT	NA	Prostate	Primary tumor
PR17PT	NA	Prostate	Primary tumor
PR18PT	NA	Prostate	Primary tumor
PR19PT	NA	Prostate	Primary tumor
SW32	SW32	Prostate	Cell line
